# Microwave-Hydrothermal Rapid Synthesis of Cellulose/Ag Nanocomposites and Their Antibacterial Activity

**DOI:** 10.3390/nano8120978

**Published:** 2018-11-27

**Authors:** Lian-Hua Fu, Qing-Long Gao, Chao Qi, Ming-Guo Ma, Jun-Feng Li

**Affiliations:** 1 Beijing Key Laboratory of Lignocellulosic Chemistry, College of Materials Science and Technology, Beijing Forestry University, Beijing 100083, China; fulianhua1990@163.com; 2 Guangdong Key Laboratory for Biomedical Measurements and Ultrasound Imaging, School of Biomedical Engineering, Health Science Center, Shenzhen University, Shenzhen 518060, China; qichao2016@sina.com; 3 College of Biological Sciences and Biotechnology, Beijing Forestry University, Beijing 100083, China; gwvorb@163.com; 4 College of Water Conservancy and Architectural Engineering, Shihezi University, Shihezi 832000, China

**Keywords:** cellulose, silver, nanocomposites, microwave-hydrothermal, antibacterial activity

## Abstract

Silver-based antimicrobial nanomaterials are considered as the most promising antibacterial agents owing to their outstanding antimicrobial efficacy and their relatively low toxicity to human beings. In this work, we report on a facile and environment-friendly microwave-hydrothermal method to prepare cellulose/Ag nanocomposites using hemicellulose as the reductant. The influences of the microwave-hydrothermal heating time and temperature, as well as the hemicellulose concentration on the formation of cellulose nanocomposites, were investigated in detail. Experimental results indicated that the hemicellulose was an effective reductant for silver ions, with higher temperature and longer heating time favoring the formation of silver with higher crystallinity and mass content in the nanocomposites. Moreover, the antimicrobial properties of the as-prepared cellulose/Ag nanocomposites were explored using Gram-positive *S. aureus* ATCC 6538 and Gram-negative *E. coli* HB 101 by both disc diffusion method and agar dilution method, and the nanocomposites showed excellent antibacterial activity. These results demonstrate that the as-prepared cellulose/Ag nanocomposites, as a kind of antibacterial material, are promising for applications in a wide range of biomedical fields.

## 1. Introduction

To fight infections and prevent spoilage caused by harmful bacteria, a lot of materials with antibacterial activity have been developed by researchers and pharmaceutical companies [1]. Among these materials, silver-based antibacterials are considered as the most promising because of their outstanding antimicrobial efficacy and broad spectrum antimicrobial properties against bacteria, viruses, and fungi. In addition, silver has been found to be nontoxic to humans in minute concentrations [1,2]. Silver nanoparticles (AgNPs) incorporated into polymer material substrates or loaded on non-metal surfaces can endow the composites with favorable optical properties, catalytic properties, and enhanced antibacterial activities [3,4,5,6]. These composite materials have been widely applied in diverse applications, such as wound dressing [7], dental material [8], cotton fabric [9], and water treatment [10]. Because the antibacterial activity of these silver-based composites have been proven to strongly depend on their size and morphology [1], the design and synthesis of these materials in which the size and morphology, as well as the stability and properties, are controlled has become a major field of interest.

In the past few years, silver-based composite materials have experienced a fast development, and numerous studies have been documented in the literature [7,11,12,13,14,15,16]. These syntheses of AgNPs often involve the reduction of an ionic salt in an appropriate medium in the presence of reductants and/or stabilizers, such as NaBH_4_, trisodium citrate, hydrazine, hydroxylamine, ascorbic acid, gelatin, polyvinylpyrrolidone (PVP), and dopamine [11,12,13,14,15]. For example, AgNPs were impregnated into bacterial cellulose using three reducing agents (hydrazine, hydroxylamine, and ascorbic acid) together with gelatin or PVP employed as colloid protectors [11]. The AgNPs were deposited on graphene oxide nanosheets using NaBH_4_ as reductant and trisodium citrate as a stabilizing agent [13]. Hebeish et al. prepared carboxymethyl cellulose hydrogels loaded with AgNPs by a two-stage method using trisodium citrate as auxiliary reductant [14]. However, the utilization of commercial reductants and/or stabilizers and the toxicity of certain agents (such as NaBH_4_, hydrazine, and hydroxylamine) pose major environmental problems and are a significant source of waste. These methods may also go against the green synthesis of AgNPs, which emphasizes three main steps: selection of solvent medium, selection of environmentally benign reducing agent, and selection of nontoxic substances for AgNP stability [15]. Hence, the development of simple and environment-friendly methods for the synthesis of AgNPs is becoming increasingly urgent.

Cellulose, one of the most abundant natural polymers and renewable resources on the earth, has been recognized as a class of renewable carbohydrate polymers that possess a number of appealing properties, such as high stiffness, low density, well-defined size and morphology, controllable surface chemistry, environmental sustainability, and anticipated low cost [17,18]. Cellulose-based nanocomposites can be obtained by filling the polymer matrix with inorganic fillers containing active molecules, such as halloysite [19,20]. Cellulose has also been extensively used as a substrate for metal nanoparticles due to the electron-rich feature of its surface OH groups, which provide cellulose with good colloidal stability in aqueous solution. The growth of metal nanoparticles can be controlled by the hydrogen-bonded network that is formed by the OH groups in the structure of cellulose [21,22]. The hemicellulose is a kind of heterogeneous polysaccharides, which can be obtained through a facile alkali extract from plants [23] and can be removed from the products by centrifugation as hemicelluloses is hydrophilic in nature. Moreover, microwave-hydrothermal heating has received considerable attention as a promising new method for the one-pot synthesis of metallic nanostructures in solutions. The use of the microwave-hydrothermal method is a viable avenue for the greener synthesis of nanomaterials and provides several desirable features, such as high reaction rate, shorter reaction time, reduced energy consumption, and environment friendliness [24,25]. Furthermore, nanostructures with smaller sizes, narrower size distributions, and a higher degree of crystallization can be obtained more consistently via microwave-hydrothermal heating than by conventional methods, such as oil bath [25].

In this work, AgNPs were incorporated into cellulose substrates by a facile and green microwave-hydrothermal method. The hemicellulose was employed as the reducing agent and removed from the products by centrifugation. The influences of the microwave-hydrothermal heating time and temperature, as well as the hemicellulose concentration on the products, were investigated in detail. The present method provides a new strategy for the preparation of cellulose/Ag nanocomposites without the use of any chemical reductants or toxic solvents. Furthermore, we evaluated the antibacterial activity of the as-prepared cellulose/Ag nanocomposites by both disc diffusion method and agar dilution method against *S. aureus* and *E. coli*. The as-prepared cellulose/Ag nanocomposites showed excellent antibacterial activity and thus are promising for applications in various biomedical fields.

## 2. Experimental Section

### 2.1. Preparation of Cellulose/Ag Nanocomposites

All chemicals used in the sample preparation were of analytical grade and used as received without further purification. The cellulose and hemicellulose were extracted from wood powder (triploid of 3-year-old *Populus tomentosa* Carr. was obtained from Shandong Province, China) followed the literature procedure [23]. For the synthesis of cellulose/Ag nanocomposites, 0.486 g of cellulose and 0.510 g of AgNO_3_ (>98.0%, purchased from Guangdong Jinhuada Chemical Reagent Co., Ltd.) were added into 30 mL deionized water under magnetic stirring. Then, 0.150 g of hemicelluloses was added into the above mixed solution under magnetic stirring. The concentration of hemicellulose in the resulting solution was 5 mg mL^−1^. After that, the resulting suspension was transferred into an autoclave (60 mL), sealed, and heated in a microwave oven (MDS-6G, Sineo, China) at 160 °C for 10 min. The product was separated by centrifugation, washed by deionized water several times and freeze-dried for further characterization. The sample obtained was labeled as S-T_160_. Other samples were prepared by similar procedures but with varying experimental parameters. The details of the preparation conditions are presented in Table 1.

### 2.2. Characterization

X-ray powder diffraction (XRD) patterns of the products were recorded in 2θ range from 10° to 70° on a Rigaku D/Max 2200-PC diffractometer operating at 40 kV with Cu Kα (λ = 1.5418 Å) radiation. Fourier transform infrared (FT-IR) spectroscopic measurements were carried out on Bruker VERTEX 70V spectrophotometer, and the spectra were recorded in the range of 4000–400 cm^−1^ at 0.4 cm^−1^ resolution and 64 scans per sample. Field emission scanning electron microscopy (FE-SEM) images and energy-dispersive X-ray spectroscopy (EDX) were recorded with Hitachi SU8010; all samples were Au-coated prior to examination. The energy of beam and working distance corresponding to FE-SEM and EDX were 5.0 kV/5.1 mm and 3.0 kV/13.2 mm, respectively. The thermal behavior of the samples was determined using thermogravimetric (TG) and differential thermal analysis (DTA) on a Shimadzu DTG-60, with a heating rate of 10 °C min^−1^ from room temperature to 600 °C under nitrogen atmosphere; the nitrogen flow rate was 50 mL min^−1^. The Ag contents in the typical samples were measured by an inductively coupled plasma (ICP) optical emission spectrometer on Horiba JY 2000-2.

### 2.3. Determination of Ag Content

The powdered cellulose/Ag nanocomposites (10 mg) were dispersed in HNO_3_ solution (0.1 M, 5 mL) by shaking at a constant rate at 37 °C for 4 h to dissolve the samples completely. After centrifugation, the supernatant was measured by ICP to determine the content of silver. The silver contents in the samples are shown in Table 1.

### 2.4. Antibacterial Activity

The antibacterial activities of the cellulose/Ag nanocomposites were investigated by the disc diffusion method against *S. aureus* ATCC 6538 as the model Gram-positive bacteria and *E. coli* HB 101 as the model Gram-negative bacteria. The concentration of *S. aureus* and *E. coli* was adjusted to approximately 1 × 10^7^ colony-forming units (CFU) mL^−1^. The cellulose/Ag nanocomposites (100 ± 1 mg per sample) were preformed into disks with uniform size (10 mm in diameter) and sterilized by autoclaving at 121 °C for 20 min. In the inhibition zone experiment, nutrient agar was poured into disposable sterilized Petri dish and solidified. Then, 100 μL of bacterial suspension was streaked over the nutrient agar and spread uniformly. After that, the circular disks of the control and the test samples were gently placed on the surface of the nutrient agar and incubated at 37 °C for 24 h. The inhibition zones, which appear as a clear area around the disks, were measured by a ruler with up to 1 mm resolution.

The minimum inhibitory concentrations (MICs) of the cellulose/Ag nanocomposites against *S. aureus* ATCC 6538 were carried out by the agar dilution method following the procedure of our previous study [26]. The cellulose/Ag nanocomposites suspension (10 mL) and casein hydrolysate agar medium (10 mL) were mixed in the culture under gently shaking. Then, 1–2 μL of bacterial suspension (with concentration of about 1 × 10^7^ CFU) was seeded on the obtained medium; the diameter of each bacterial suspension ring was about 5~8 mm. Then, the culture was inverted and incubated at 35 °C for 24 h. The concentrations of the cellulose/Ag nanocomposite suspension used for preliminary screening were 80, 40, 20, 10, 5, 2.5, 1.25, and 0.625 mg mL^−1^, and the concentrations were 10, 4, 2, 1, 0.5, and 0.25 mg mL^−1^ for the second screening. The culture plate containing casein hydrolysate agar medium was seeded with the bacterial suspension and used as the positive control. The negative control was prepared by the culture plate containing only casein hydrolysate agar medium. Three parallel experiments were performed for each measurement.

## 3. Results and Discussion

**Figure 1:**
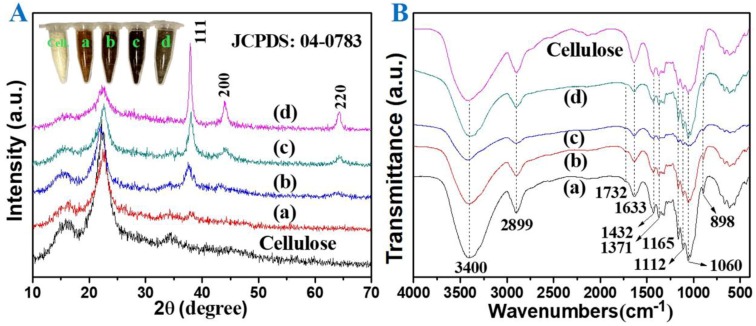
(**A**) XRD patterns and (**B**) FT-IR spectra of samples. (**a**) S-T_120_, (**b**) S-T_140_, (**c**) S-T_160_, and (**d**) S-T_180_. The inset on the top left corner of the XRD patterns (**A**) is the optical photograph of the cellulose and the products.

The XRD patterns and FT-IR spectra of the products prepared by the microwave-hydrothermal method at 120–180 °C for 10 min with hemicellulose concentration of 5 mg mL^−1^ are shown in Figure 1. The optical photographs (the inset of Figure 1A) showed that the color of the product gradually deepened with the increase in heating temperature from 120 to 160 °C, while the color of the sample turned into gray when the temperature further increased to 180 °C. In Figure 1A, the diffraction peaks around 2θ = 15.6° and 22.5° can be attributed to cellulose (JCPDS 03-0289), and the other diffraction peaks can be indexed to a single phase of silver with cubic structure (JCPDS 04-0783). The silver content of sample S-T_120_ was measured to be 2.2% by ICP (Table 1), and only a weak diffraction peak around 2θ = 38.0° corresponding to the crystal plane (111) of silver was observed in the XRD pattern (Figure 1Aa). When the heating temperature was increased to 140 °C, the content of silver increased to 4.7% (Table 1), and the intensity of the peak around 2θ = 37.6° became stronger (Figure 1Ab). When the temperature increased to 160 °C, the peak at 2θ = 38.1° further strengthened, and the peaks attributed to crystal planes of (200) and (220) could be observed (Figure 1Ac), indicating that the well-crystallized phase of silver could be obtained at 160 °C for a short period of time (10 min). In our previous study, only a weak peak at 2θ = 37.9° was observed in the product prepared under a similar heating method without usage of hemicellulose at 160 °C for 60 min [27]. This result indicated that the hemicellulose was an effective reductant for silver ions. The sharp peaks of silver were observed in the sample prepared at 180 °C (Figure 1Ad), suggesting the higher reaction temperature increased the crystallinity of silver. From Figure 1A and Table 1, one can conclude that the higher temperature favored the formation of silver with higher crystallinity. In addition, the high temperature was conducive to the reductibility of hemicellulose to silver ions, where the contents of silver were 2.2, 4.7, 7.9, and 11.4%, corresponding to the samples prepared at 120, 140, 160, and 180 °C, respectively.

In the FT-IR spectra (Figure 1B), the absorptions at 3400 cm^−1^ and 1371 cm^−1^ can be attributed to the stretching and bending vibrations of –OH groups, and the peak located at 1633 cm^−1^ can be ascribed to the absorbed moisture in the nanocomposites. The peak at 2899 cm^−1^ originated from the stretching modes of CH_2_ and C–H groups. The symmetric bending of CH_2_ and C–OH skeletal vibration were seen at 1432 cm^−1^ and 1112 cm^−1^, respectively. The characteristic of β-glycosidic linkages between glucose units was located at 1165 cm^−1^ and 895 cm^−1^, and the band at 1052 cm^−1^ corresponded to the vibration of C–O–C pyranose ring skeletal of cellulose. It is notable that a weak peak at 1732 cm^−1^ identified the presence of carbonyl groups, which is characteristic of hemicelluloses [28]. This result might have resulted from the residue of hemicellulose because hemicelluloses can form hydrogen bonding with cellulose [23]. In Figure 1B, it can be seen that all the as-prepared samples displayed typical bands of cellulose, indicating that the original structure of cellulose can be well maintained after microwave-hydrothermal treatment.

**Figure 2:**
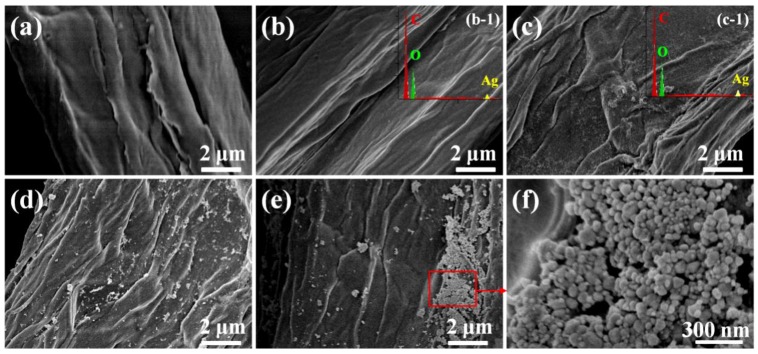
FE-SEM images of cellulose substrate and cellulose/Ag nanocomposites: (**a**) cellulose, (**b**) S-T_120_, (**c**) S-T_140_, (**d**) S-T_160_, (**e**,**f**) S-T_180_. The insets on the top right corner of the images are the EDX spectra (plane scan analysis) of samples (**b-1**) S-T_120_ and (**c-1**) S-T_140_.

It is well known that the surface of cellulose is terminated with OH groups, which was also proven in our study with FT-IR spectra (Figure 1B), where all the four samples exhibited broad OH stretching band over 3600 and 3000 cm^−1^ [29]. In the reaction process, the silver ions could have firstly been absorbed in the OH functional groups of cellulose through electrostatic interactions, and these absorbed silver ions in turn served as the seeds for the deposition of reduced silver [30]. In addition, the silver nuclei can be formed more rapidly at higher temperature, leading to the different levels of AgNPs deposited on the cellulose at different temperatures, as shown in FE-SEM images (Figure 2). In Figure 2b–f, it can be seen that the morphologies of the cellulose substrates were well maintained after microwave-hydrothermal process (compared with Figure 2a), but the surface became coarse. It was difficult to observe AgNPs in the sample prepared at 120 °C (Figure 2b), but the EDX spectrum (Figure 2b-1) displayed that the product mainly consisted of C, O, and minor amount of Ag. When the temperature increased to 140 °C, the sample composed of C, O, and Ag (Figure 2c-1), and a few AgNPs appeared in the horizon (Figure 2c). These results were consistent with XRD patterns (Figure 1A). When the temperature increased to 160 °C, the cellulose substrate was covered by homogeneously dispersed AgNPs (Figure 2d). However, the AgNPs were partially agglomerated when the temperature increased to 180 °C (Figure 2e,f). Combined with the XRD patterns and FT-IR spectra, one can draw the following conclusions: (1) The hemicellulose was an effective reductant for silver ions. (2) The higher temperature favored the formation of silver with higher crystallinity. (3) The microwave-hydrothermal temperature had a significant effect on the dispersity of AgNPs, and AgNPs with good dispersity could be obtained at 160 °C.

**Figure 3:**
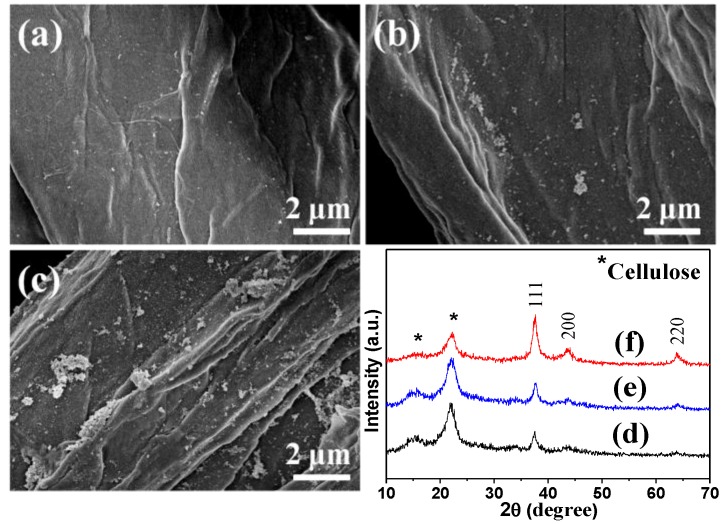
(**a**–**c**) FE-SEM images and (**d**–**f**) XRD patterns of samples (**a**,**d**) S-H_0.5_, (**b**,**e**) S-H_1_, and (**c**,**f**) S-H_10_.

In order to investigate the influence of hemicellulose concentrations on the nanocomposites, the products were prepared with different concentrations of hemicellulose. In the XRD patterns (Figure 3d–f), the peaks of cellulose were observed at 2θ = 15.4° and 22.1° (marked with *), and the other peaks could be indexed to crystallized silver (JCPDS 04-0783). The peak intensities of silver increased with increase in hemicellulose concentration, implying that higher hemicellulose concentration could lead to higher crystallinity of silver. The results from FE-SEM images (Figure 3a–c) were similar to Figure 2b–e. Only a few AgNPs were observed for the sample prepared using hemicellulose concentration of 0.5 mg mL^−1^ (Figure 3a). The number of AgNPs increased when the hemicellulose concentration increased to 1 mg mL^−1^ (Figure 3b). When the concentration further increased to 10 mg mL^−1^, the AgNPs were partially agglomerated (Figure 3c). In addition, as seen in Table 1, the contents of silver increased from 3.1% (with hemicellulose concentration of 0.5 mg mL^−1^) to 10.5% (with hemicellulose concentration of 10 mg mL^−1^). It is well known that the synthesis of silver crystals involves nucleation, growth, and aggregation processes. Based on the above results, it is important to choose rational hemicellulose concentration to prepare the cellulose/Ag nanocomposites with appropriate silver content and dispersity.

**Figure 4:**
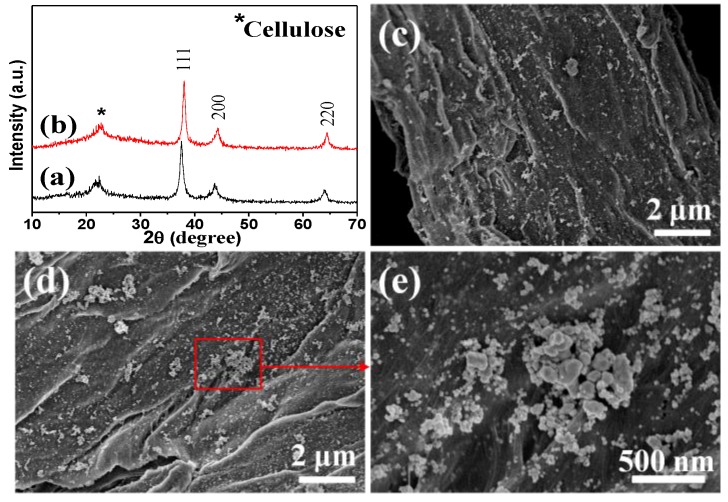
(**a**,**b**) XRD patterns and (**c**–**e**) FE-SEM images of samples (**a**,**c**) S-t_30_ and (**b**,**d**,**e**) S-t_60_.

Based on the above results, the influence of microwave-hydrothermal heating time on the nanocomposites was also examined, and the products were characterized by XRD and FE-SEM, as shown in Figure 4. Compared with the sample prepared for 10 min (Figure 1Ac), the peak of cellulose weakened (only a small peak at 2θ = 22.7° was observed), and the peak intensities of silver strengthened for the samples prepared for 30 (Figure 4a) and 60 min (Figure 4b), suggesting the higher crystallinity of silver. Moreover, the FE-SEM images showed that the cellulose substrates were covered by densely dispersed AgNPs (Figure 4c,d), and agglomerated AgNPs were also observed from the high magnification image (Figure 4e). The contents of silver were 15.4 and 23.1% (Table 1), corresponding to the samples prepared for 30 and 60 min, respectively. These results showed that the microwave-hydrothermal heating time had critical influence on the preparation of AgNPs. It is supposed that more silver ions could grasp the OH functional groups of cellulose with the prolong reaction time, which were reduced by hemicellulose in the aqueous solution. Moreover, the longer reaction time contributed to the growth of AgNPs with bigger size, which was proven by the size of AgNPs calculated according to the Scherrer formula (Equation (1)).

(1) $$D=\frac{K\lambda }{\beta \;\cos\;\theta }$$

where λ is 1.5418 Å, *β* is half peak width, and K is constant and assigned as 0.89 here; the peak of (111) was chosen to calculate the size of AgNPs. The sizes of AgNPs in the products prepared for 10, 30, and 60 min were 13.5, 15.3, and 16.0 nm, respectively. In our previous study, a similar result was also obtained using fructose as the reducing agent through conventional hydrothermal method for 3 to 12 h [31].

**Figure 5:**
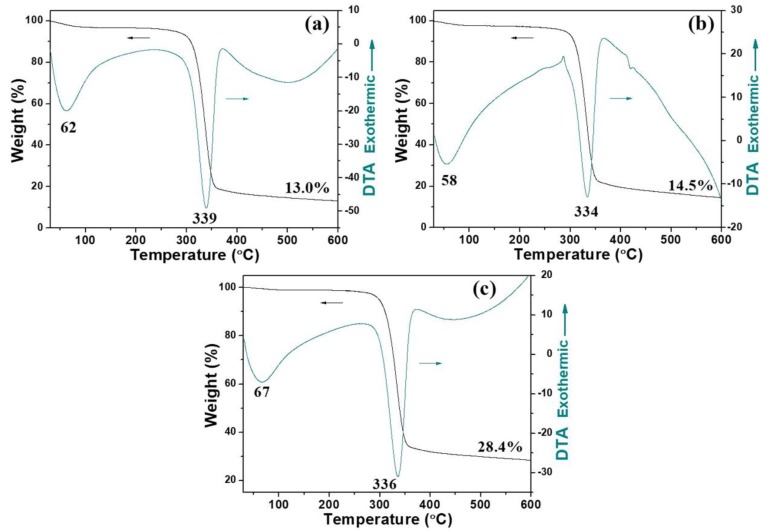
Thermogravimetric (TG) and differential thermal analysis (DTA) curves of the products prepared with hemicellulose concentration of 5 mg mL^−1^ at 160 °C for (**a**) 10 min, (**b**) 30 min, and (**c**) 60 min.

The thermal stability of the as-prepared cellulose/Ag nanocomposites was investigated by TG and DTA under nitrogen atmosphere (Figure 5); the TG and DTA parameters are summarized in Tables S1 and S2. Figure 5a shows the TG and DTA curves of the sample prepared with hemicellulose concentration of 5 mg mL^−1^ by the microwave-hydrothermal method at 160 °C for 10 min. Obviously, the weight loss of the sample was mainly in the range of 300–400 °C, which was due to the degradation of cellulose substrate. The slight weight loss around 100 °C was assigned to the absorbed moisture. The DTA curve showed two endothermic peaks located around 62 and 339 °C (Figure 5a, Table S2), which fit well with the weight losses in the TG curve. The weight losses of the samples prepared for 30 and 60 min (Figure 5b,c) were similar, and the final weight losses in the temperature range investigated were about 87.0, 85.5, and 71.6% (Table S1), corresponding to the sample prepared for 10, 30, and 60 min, respectively. Combined with the silver contents of 7.9% (10 min), 15.4% (30 min), and 23.1% (60 min) from ICP analysis (Table 1), it was concluded that the microwave-hydrothermal heating time did not have a big influence on the thermal stability of the products.

**Figure 6:**
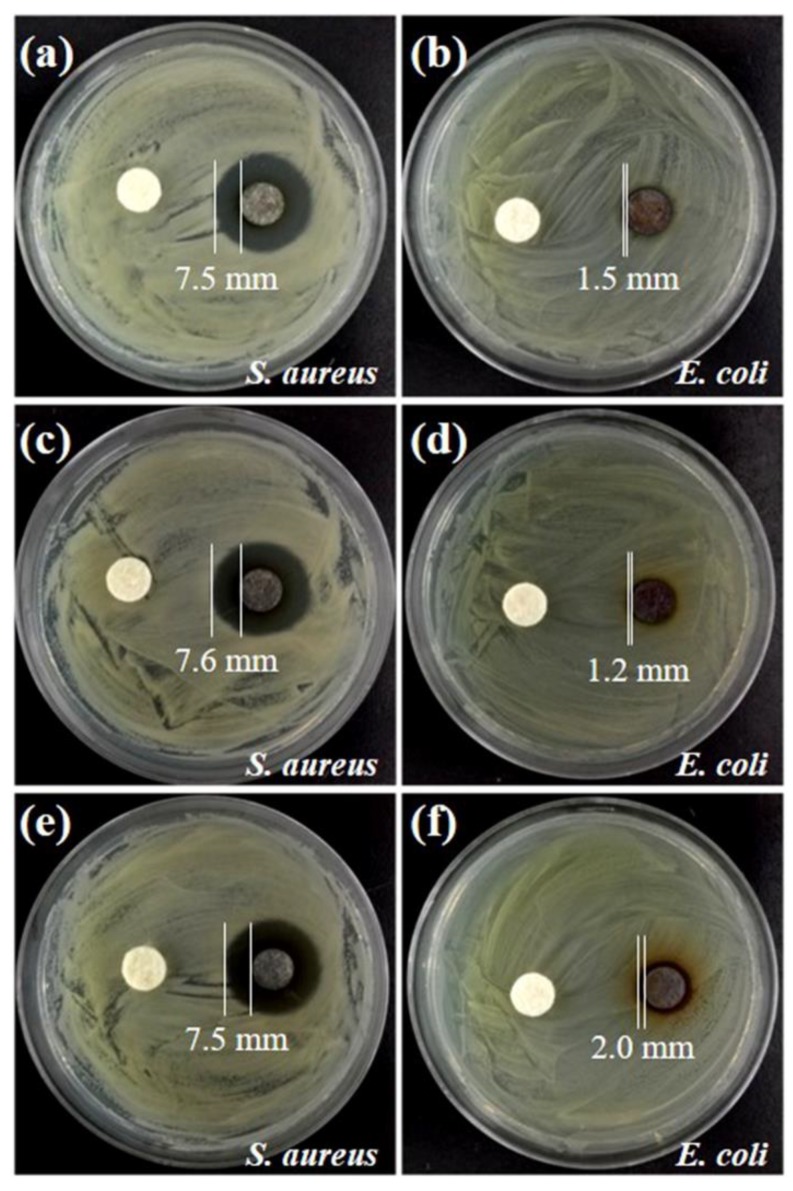
The antibacterial activities of the products prepared with hemicellulose concentration of 5 mg mL^−1^ at 160 °C for (**a**,**b**) 10 min, (**c**,**d**) 30 min, and (**e**,**f**) 60 min. The samples in the left of the cultures are pure cellulose, and those in the right are nanocomposites.

The antibacterial activities of the cellulose/Ag nanocomposites prepared with hemicellulose concentration of 5 mg mL^−1^ by the microwave-hydrothermal method at 160 °C for different times were investigated against *S. aureus* ATCC 6538 and *E. coli* HB 101 by disc diffusion method, and pure cellulose was used as control. The results are shown in Figure 6. No inhibition zones could be observed for the pure cellulose, indicating that the pure cellulose had no antibacterial activities. All the samples prepared for 10, 30, and 60 min exhibited obvious inhibition zones for both *S. aureus* and *E. coli*, as shown in Figure 6. The inhibition zones against *S. aureus* were 7.5, 7.6, and 7.5 mm, corresponding to the samples prepared for 10, 30, and 60 min (Figure 6a,c,e), respectively. These data are larger than those reported in a previous work in which the inhibition zones for *S. aureus* were only 1.0–2.0 mm [32]. In Figure 6b,d,f, it can be seen that the inhibition zones against *E. coli* were 1.5, 1.2, and 2.0 mm, corresponding to the products prepared for 10, 30, and 60 min, respectively. It is clear that the inhibition zones against *S. aureus* (7.5–7.6 mm) were larger than *E. coli* (1.2–2.0 mm), which might have been be caused by the difference in the susceptibility of Gram-positive and Gram-negative bacteria to the nanocomposites. The additional outer membrane and lower content of peptidoglycan in the cell membranes of Gram-negative bacteria could make *E. coli* less susceptible to the cellulose/Ag nanocomposites [33,34], leading to the smaller inhibition zones. Similar results were also observed in our previous study [26]. Although the inhibition zones against *E. coli* were 1.2–2.0 mm for the as-prepared cellulose/Ag nanocomposites, it is comparable to the cellulose/Ag/AgCl hybrids prepared at 160 °C for 12 h through hydrothermal method [32]. The results of antibacterial experiments showed that the as-prepared cellulose/Ag nanocomposites had good antibacterial activities against both *S. aureus* (Gram-positive) and *E. coli* (Gram-negative) bacteria. In addition, it is worth noting that there was no big difference in the inhibition zones for the three samples against *S. aureus* (7.5, 7.6, and 7.5 mm), while there was a little difference for those against *E. coli* (1.5, 1.2, and 2.0 mm). Previous literatures have reported that the antimicrobial properties of AgNPs are affected by their nanometer size, morphology, specific surface area, and the amount of silver [1,12], with the smaller particles having higher activities on the basis of equivalent silver mass content [35]. In this study, both the size and silver content increased with prolonged microwave-hydrothermal heating time. The size (mass content) of AgNPs in the samples prepared for 10, 30, and 60 min were 13.5 nm (7.9%), 15.3 nm (15.4%), and 16.0 nm (23.1%), respectively. Therefore, it is proposed that the similar inhibition zones against *S. aureus* might have resulted from the synergistic effects of the size and content of silver. Regarding *E. coli*, the differences in the inhibition zones might have been affected by factors other than the size and content of silver, such as the difference in cell walls between *S. aureus* and *E. coli* and the releasing behavior of silver [30]. In addition, the dispersity of AgNPs on cellulose substrate might have also affected the antimicrobial properties of the products as the aggregation of AgNPs might have diminished or lost the typical properties associated with their nanoscale size [12]. From the FE-SEM images (Figure 4c–e), it was observed that AgNPs were homogeneously dispersed on cellulose substrate for the sample prepared for 10 min (Figure 2d), while densely dispersed and agglomerated AgNPs were observed in the samples prepared for 30 and 60 min (Figure 4c–e). Further study should be conducted to investigate the intrinsic mechanism of the effects on the antimicrobial properties so as to provide more details for application of the as-prepared cellulose/Ag nanocomposites.

**Figure 7:**
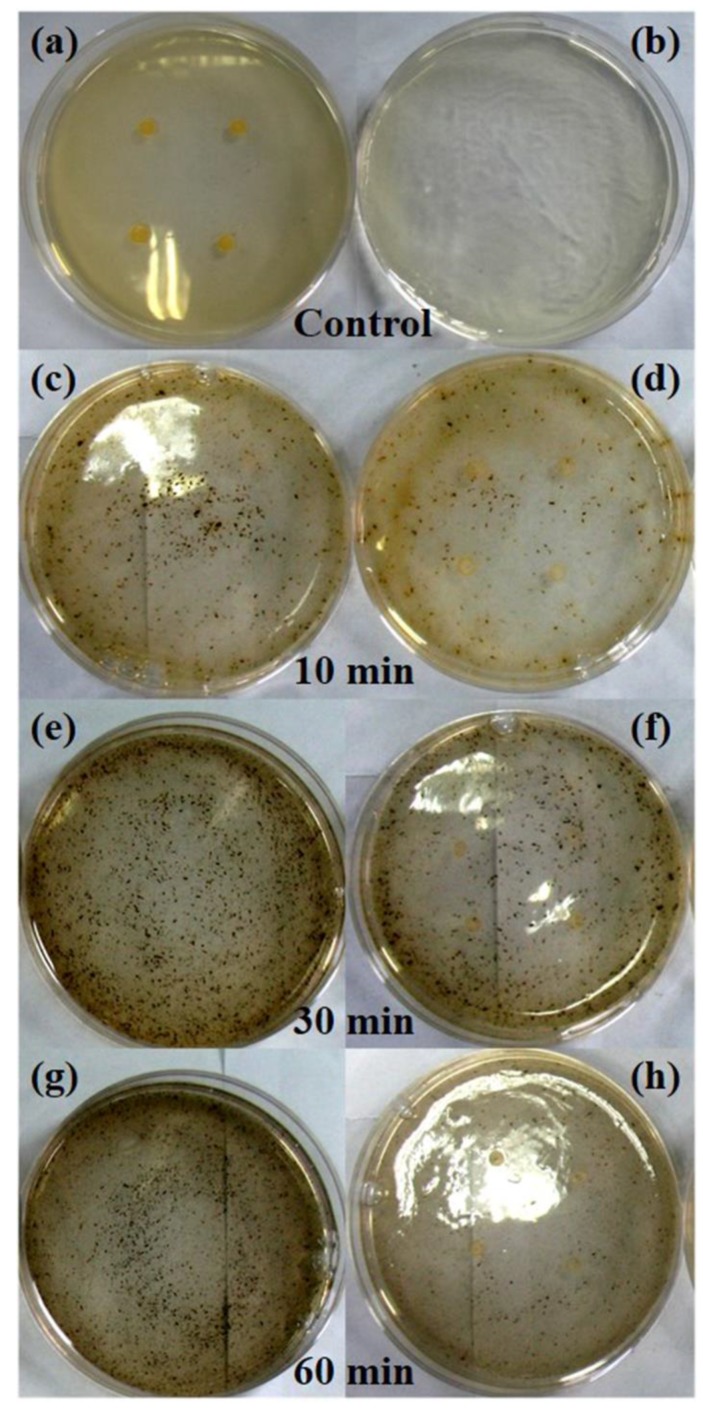
The MICs of the (**a**) positive and (**b**) negative controls and the products (**c**–**h**) prepared with hemicellulose concentration of 5 mg mL^−^^1^ at 160 °C for different times against *S. aureus*. The concentrations of the nanocomposites suspension added in the cultures were 1, 0.5, 2, 1, 2, and 1 mg mL^−^^1^, corresponding to (**c**–**h**), respectively.

The minimum inhibition concentration (MIC) is known as the lowest concentration of the applied antibacterial material that can inhibit the growth of an organism; the material with high antibacterial activity would exhibit lower MICs. In this study, the MICs of the products prepared by the microwave-hydrothermal method with hemicellulose concentration of 5 mg mL^−1^ at 160 °C for different times were investigated against *S. aureus* ATCC 6538 by the agar dilution method. The optical photographs of the second screening are shown in Figure S1. The MICs were found to be 1, 2, and 2 mg mL^−1^, corresponding to the samples prepared for 10, 30, and 60 min, respectively. Enlarged images of the controls and the neighboring concentrations of cellulose/Ag nanocomposites that could and could not inhibit the growth of *S. aureus* are also shown in Figure 7. All the three samples were found to be more effective than the holocellulose/Ag nanocomposites prepared by hydrothermal method for 12 h in our previous study in which the MICs were 2.5–20 mg mL^−1^ against *S. aureus* [26]. The enhanced antimicrobial properties of the cellulose/Ag nanocomposites obtained in the present work might have benefitted from the releasing behavior of silver in the nanocomposites. Previous studies have revealed that cellulose can be used as a substrate for metal nanoparticles due to the electron-rich feature of its surface OH functional groups, which provide cellulose with good colloidal stability in aqueous solution. Cellulose can stabilize nanoparticles through the electrostatic interaction of their OH functional groups with the nanoparticles [12,21,22]. In the present work, cellulose served as the matrix for AgNPs, and the surface OH functional groups of cellulose could not only interact with AgNPs through electrostatic interactions but also form intra- and intermolecular hydrogen bonds in the structure of cellulose. It is thought that the AgNPs bound to the cellulose networks structure, which controlled the release of silver ions [32], and thus the AgNPs were released in a sustained way. The present study clearly indicates that the cellulose/Ag nanocomposites prepared by the microwave-hydrothermal method show excellent antibacterial activity against both Gram-positive and Gram-negative bacteria strains. Combining all the beneficial qualities, the as-prepared cellulose/Ag nanocomposites may have potential for use in a wide range of biomedical applications.

## 4. Conclusions

In summary, a facile, rapid, surfactant-free and environment-friendly strategy was developed for the synthesis of cellulose/Ag nanocomposites through the microwave-hydrothermal method using hemicellulose as the reductant. Experimental results revealed that hemicellulose was an effective reductant for silver ions, and the size, mass content, and dispersity of AgNPs on the cellulose substrates were related to the microwave heating time and temperature as well as the hemicellulose concentration. The as-prepared cellulose/Ag nanocomposites possessed excellent antimicrobial properties against both Gram-positive (*S. aureus*) and Gram negative (*E. coli*) bacteria. Thus, the as-prepared cellulose/Ag nanocomposites are promising for applications in various biomedical fields.

## Figures and Tables

**Table 1:** Experimental conditions for the preparation of typical samples using hemicellulose as reductant by the microwave-hydrothermal method.

Sample No.	Hemicellulose (mg mL^−1^)	Temperature (°C)	Time (min)	Ag Content (%)
**S-T_120_**	5	120	10	2.2
**S-T_140_**	5	140	10	4.7
**S-T_160_**	5	160	10	7.9
**S-T_180_**	5	180	10	11.4
**S-H_0.5_**	0.5	160	10	3.1
**S-H_1_**	1	160	10	3.7
**S-H_10_**	10	160	10	10.5
**S-t_30_**	5	160	30	15.4
**S-t_60_**	5	160	60	23.1

## Supplementary Materials

The following are available online at http://www.mdpi.com/2079-4991/8/12/978/s1, Figure S1: The second screening of MICs for the products prepared with hemicellulose concentration of 5 mg mL^−1^ at 160 °C for different times against *S. aureus*, Table S1: The weight losses (TG) of the sample at different temperature corresponding to Figure 5, Table S2: The endothermic peaks (DTA) of the sample corresponding to Figure 5.
