# Supplementary material for: Microwave-Hydrothermal Rapid Synthesis of Cellulose/Ag Nanocomposites and Their Antibacterial Activity
Source: Nanomaterials (Basel). 2018 Nov 27;8(12):978. doi: 10.3390/nano8120978 (PMC6316342; doi:10.3390/nano8120978)
Supplement: Supplementary file 1 [file nanomaterials-08-00978-s001.pdf]

Support information

Microwave-Hydrothermal Rapid Synthesis of Cellulose/Ag Nanocomposites and Their Antibacterial Activity

Lian-Hua Fu [1,2], Qing-Long Gao [3], Chao Qi [2], Ming-Guo Ma [1,*] and Jun-Feng Li [4,*]

1 Beijing Key Laboratory of Lignocellulosic Chemistry, College of Materials Science and Technology, Beijing Forestry University, Beijing 100083, China; fulianhua1990@163.com
2 Guangdong Key Laboratory for Biomedical Measurements and Ultrasound Imaging, School of Biomedical Engineering, Health Science Center, Shenzhen University, Shenzhen 518060, China; qichao2016@sina.com
3 College of Biological Sciences and Biotechnology, Beijing Forestry University, Beijing 100083, China;
4 College of Water Conservancy and Architectural Engineering, Shihezi University, Shihezi 832000, China; ljf205@shzu.edu.cn
* Correspondence: mg_ma@bjfu.edu.cn (M.-G.M.); ljf205@shzu.edu.cn (J.-F.L.); Tel.: +86-10-62337250 (M.-G.M.); Fax: +86-10-62336903 (M.-G.M.)

Table S1. The weight losses (TG) of the sample at different temperature corresponding to **Figure 5**.

Sample	Weight Loss at 100 °C	Weight Loss at 340 °C	Weight Loss at 600 °C
**a**	3.0%	58.2%	87.0%
**b**	2.3%	64.8%	85.5%
**c**	1.0%	51.6%	71.6%

Table S2. The endothermic peaks (DTA) of the sample corresponding to **Figure 5**.

Sample	Endothermic Peak 1	Endothermic Peak 2
**a**	62 °C	339 °C
**b**	58 °C	334 °C
**c**	67 °C	336 °C

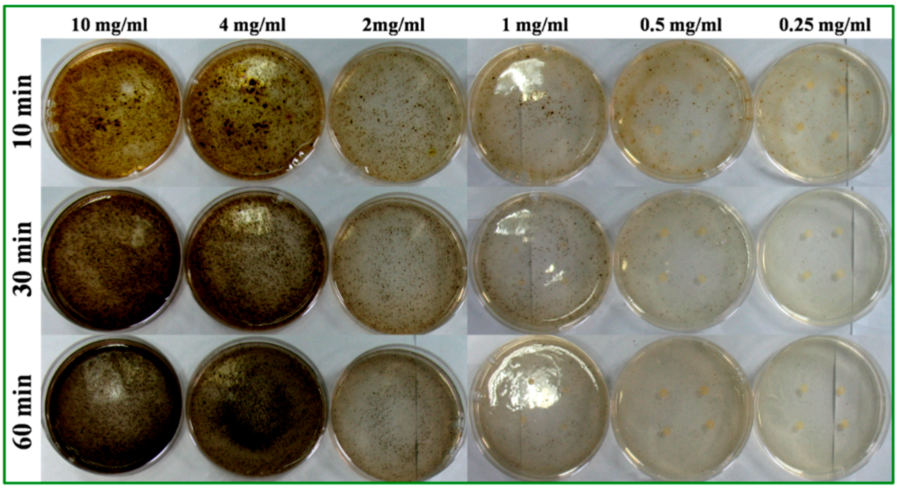

Figure S1. The second screening of MICs for the products prepared with hemicellulose concentration of 5 mg mL^{-1} at 160 °C for different times against *S. aureus*.
